# Pursestring encirclement before endoscopic submucosal excavation of a cecal submucosal tumor

**DOI:** 10.1055/a-2173-7284

**Published:** 2023-11-14

**Authors:** Jinfeng Zhou, Jiangguo Zhang, Xiaoyin Zhang

**Affiliations:** Department of Gastroenterology, National Clinical Research Center of Infectious Disease, The Third People’s Hospital of Shenzhen, The Second Affiliated Hospital of Southern University of Science and Technology, Shenzhen, China

Endoscopic submucosal excavation (ESE) has been successfully applied to the resection of gastric submucosal tumors (SMTs) 1 2 3 4 , but rarely to colonic SMTs because of the inevitability of perforation and subsequent peritonitis. The perforation is usually difficult to close quickly using titanium clips, owing to the collapse of the operational field of view, which is hard to restore by insufflation of carbon dioxide. Here, we report a novel method, named “pursestring encirclement before ESE” (PSE-ESE) ( Video 1 ), for the endoscopic excavation of a cecal SMT. With the use of PSE-ESE, closure of the colonic perforation became quick and easy.

**Video 1** Pursestring encirclement before endoscopic submucosal excavation of a cecal submucosal tumor in a 50-year-old man.


A 50-year-old man underwent a screening colonoscopy in which an 8-mm SMT was discovered in the cecum ( Fig. 1 a ). Endoscopic ultrasound examination revealed that the tumor was originating from the serosal layer. Firstly, the pursestring encirclement, using a nylon thread (HX-400U-30; Olympus) and three titanium clips (ROCC-D-26–195; Micro-Tech Nanjing), was established around the tumor, with the clips placed 5–8 mm away from the tumor margin ( Fig. 1 b ). Lifting solution, consisting of sodium hyaluronate, saline, and indigo carmine, was then injected submucosally ( Fig. 1 c ), and this was followed by a mucosal incision of about 12 mm, within the pursestring encirclement ( Fig. 1 d ). The SMT was excavated by carefully dissecting the submucosal tissues, muscularis propria, and then the serosal layer ( Fig. 1 e ), before being resected en bloc and removed ( Fig. 1 f ). A Woodpecker knife (EK-425D; Anrei) was used in the ESE procedure. A small hole of 4 mm could be seen after the ESE procedure ( Fig. 1 g ), but this was easily closed within a minute by tightening the ring of encirclement ( Fig. 1 h ). No complications such as abdominal pain or fever were observed after the endoscopic surgery. Pathologic examination revealed that the tumor was a lipoma.

**Fig. 1:**
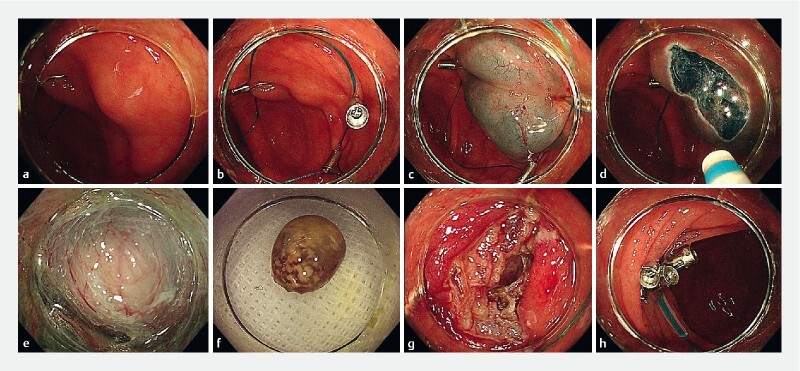
Images of the pursestring encirclement before endoscopic submucosal excavation (PSE-ESE) procedure being performed for a cecal submucosal tumor showing: **a** an endoscopic view of the submucosal tumor in the cecum; **b** the pursestring, consisting of a nylon thread and clips, encircling the tumor; **c** the appearance following injection of the lifting solution; **d** the mucosal incision being made; **e** excavation of the tumor by dissection of the submucosal tissue and muscularis propria; **f** the tumor following its removal; **g** the tissue defect and perforation after ESE; **h** the defect after closure of the perforation by tightening of the ring of encirclement.

PSE-ESE can facilitate the closure of a perforation and avoid peritonitis. It is therefore a feasible, effective, and safe treatment for colonic SMTs.

Endoscopy_UCTN_Code_TTT_1AQ_2AD

## Notes

Endoscopy E-Videos

https://eref.thieme.de/e-videos
*E-Videos* is an open access online section of the journal *Endoscopy* , reporting on interesting cases and new techniques in gastroenterological endoscopy. All papers include a high-quality video and are published with a Creative Commons CC-BY license. Endoscopy E-Videos qualify for HINARI discounts and waivers and eligibility is automatically checked during the submission process. We grant 100% waivers to articles whose corresponding authors are based in Group A countries and 50% waivers to those who are based in Group B countries as classified by Research4Life (see: https://www.research4life.org/access/eligibility/ ). This section has its own submission website at https://mc.manuscriptcentral.com/e-videos
